# Multi-Level 3D Surgery for Obstructive Sleep Apnea: Could It Be the Future?

**DOI:** 10.3390/jcm12134173

**Published:** 2023-06-21

**Authors:** Angelo Eplite, Claudio Vicini, Giuseppe Meccariello, Giannicola Iannella, Antonino Maniaci, Angelo Cannavicci, Francesco Moretti, Fabio Facchini, Tommaso Mazzocco, Giovanni Cammaroto

**Affiliations:** 1 Department of Biomedical and Clinical Sciences “Luigi Sacco”, University of Milan, Via GB Grassi 74, 20154 Milan, Italy; 2 Department of Head-Neck Surgery, Otolaryngology, Head-Neck and Oral Surgery Unit, Morgagni Pierantoni Hospital, Via Carlo Forlanini 34, 47121 Forli, Italy; claudio@claudiovicini.com (C.V.); drmeccariello@gmail.com (G.M.); a.cannavicci.md@gmail.com (A.C.); giovanni.cammaroto@hotmail.com (G.C.); 3 Department of ‘Organi di Senso’, University “Sapienza”, Viale dell’Università 33, 00185 Rome, Italy; giannicola.iannella@uniroma.it; 4 Department of Medical and Surgical Sciences and Advanced Technologies “GF Ingrassia”, ENT Section, University of Catania, Piazza Università 2, 95100 Catania, Italy; tonymaniaci@hotmail.it; 5 Department ENT & Audiology, University of Ferrara, Via Savonarola 9, 44121 Ferrara, Italy; francesco.moretti@unife.it (F.M.); fabio.facchini@unife.it (F.F.); tommaso.mazzocco@unife.it (T.M.)

**Keywords:** 3D surgery, coblator, 3D tongue base resection, 3D barbed reposition pharyngoplasty

## Abstract

(1) Background: Obstructive sleep apnea (OSA) is the most common sleep-related breathing disorder and is characterized by recurrent episodes of complete or partial obstruction of the upper airway, leading to reduced or absent breathing during sleep. A nocturnal upper airway collapse is often multi-levelled. The aim of this communication is to describe a 3D multi-level surgery setting in OSA pathology, introducing new surgical approaches, such as 4K-3D endoscopic visualization for the tongue base approach with the aid of a coblator and exoscopic visualization in the palatal approach. (2) Methods: Seven patients affected by OSA underwent 3D Barbed Reposition Pharyngoplasty (BRP) surgery associated with transoral coblation tongue base reduction and nose surgery. (3) Results: No patients experienced intra-operative, post-operative or delayed complications. For OSA multi-level 3D surgery, it took less than 2 h: the median 3D system setting time was 12.5 ± 2.3 min; the overall procedure time was 59.3 ± 26 min. (4) Conclusions: The use of the 4K-3D endoscope and coblator for tongue base resectioning and of the 3D exoscope for lateral pharyngoplasty represents an excellent system in multi-level OSA related surgery that could reduce the time and the costs compared to those of robotic surgery.

## 1. Introduction

Obstructive sleep apnea (OSA) syndrome is a respiratory sleep disorder characterized by partial or complete recurrent episodes of upper airway collapses that occur during the night [1]. A nocturnal upper airway collapse is often multi-levelled. Several surgical procedures have been developed in recent years to correct retrolingual and retropalatal collapses [2]. In the last 15 years, TORS has been widely used for the resectioning of excess baselingual lymphatic tissue, which causes secondary epiglottis, as well as in epiglottoplasty in cases of primary epiglottis [3]. The use of robotic surgery and innovative surgery on the soft palate called “Barbed Reposition Pharyngoplasty” (BRP) represent the fundamental points of multi-level surgery on OSA patients [4].

Recently, our group introduced new surgical approaches using, i.e., 4K-3D endoscopic visualization for the tongue base approach and exoscopic visualization for the palatal approach. Hereafter, we describe the technique of a multi-level surgery setting and report on its feasibility and safety.

## 2. Material and Methods

This study was approved by the Ethics Committee of the Morgagni-Pierantoni Hospital (rif. 34/2022) on 10 March 2022. Prior to the study, all individual participants included signed an informed consent form.

Each patient (of the seven recruited) was placed in the supine position on the operating table. In multi-level surgery for obstructive sleep apnea, including septal correction, tongue base and palatal surgeries, general anesthesia was given via orotracheal intubation.

**Figure 1:**
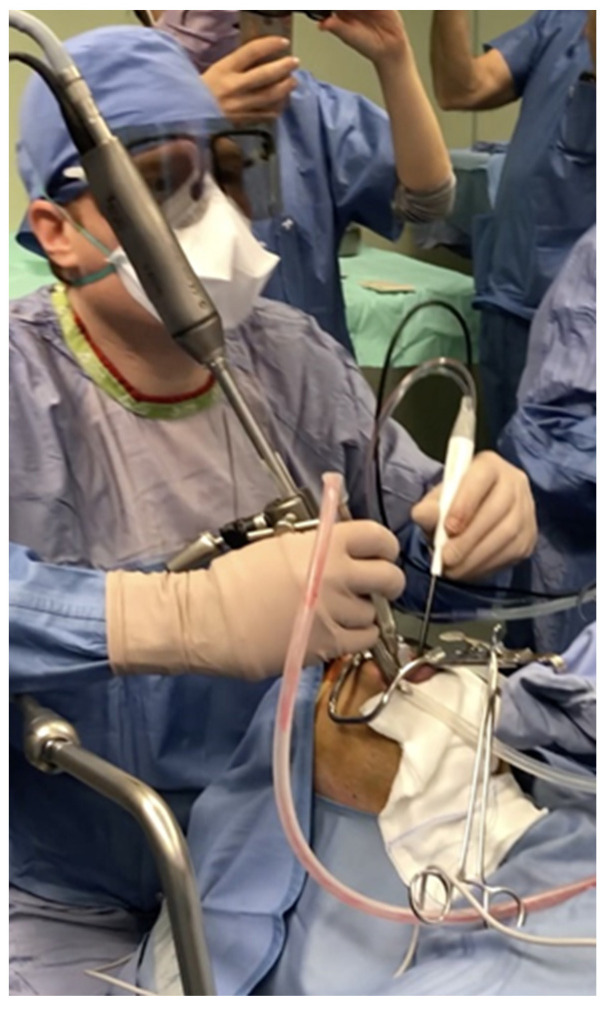
The 4K-3D videoendoscope with 10 mm diameter and 30° field of view used for the base tongue approach was assembled on a mechanical holder, and then attached to the bed using an autostatic arm. The first surgeon stood near the patient’s head, facing a 3D monitor placed beside the operating table toward its end in order to visualize the anatomical structures in a defined way.

A 4K-3D videoendoscope with a 10 mm diameter and a 30° field of view for the tongue base approach was assembled on a mechanical holder, and then attached to the bed using an autostatic arm (Figure 1). The 3D exoscopic system for the BRP (after palatine tonsillectomy) was fixed to the Versacrane™ holding system, which was positioned on one side of the surgeon; the exoscope and the Versacrane™ holding arm were connected to a clamping jaw.

All patients were in a “sniffing position” (neck flexed and head extended): the exposure of the tongue base was achieved with a single silk suture in the oral tongue, which was tractioned outside the mouth. There are several types of mouth gags and retractors, depending on the type of procedure. We used the Davis Meyer mouth gag, which was suspended by an ordinary Mayo stand. These mouth gags come with two types of tongue blades. Russel Davis blades with a groove for the endotracheal tube allow the tube to be fixed along the midline, and they are typically used for tonsillectomies and palatal surgery. Flat blades, instead, have a lower profile and allow there to be more space in the oral cavity, but require an endotracheal tube to be fixed to the side of the oral cavity. Flat blades also have two suction pumps for smoke evacuation.

**Figure 2:**
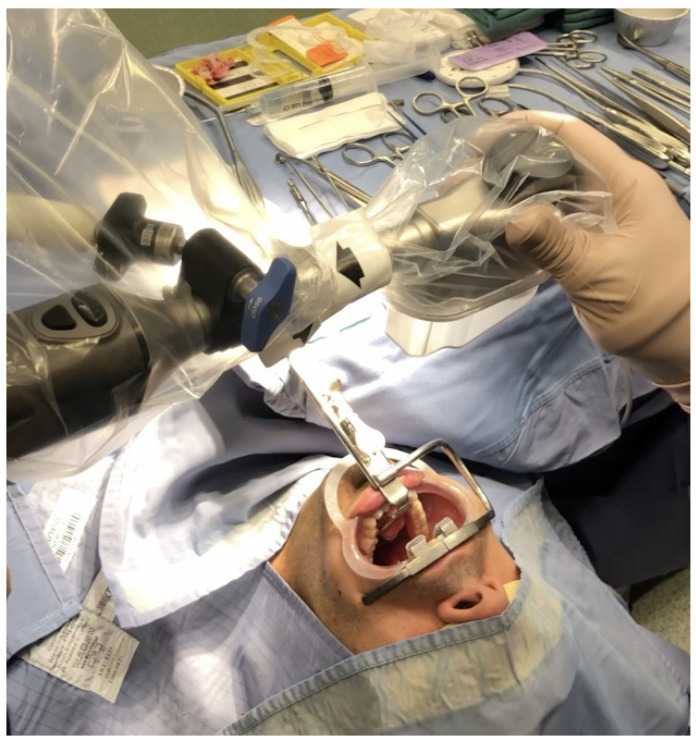
Storz Crowe–Davis mouth gag with a wide and hollow blade was placed and suspended using a lifting Mayo stand. A plastic cheek retractor was also used to make wider the oral opening and protect oral commissure. The exoscope was positioned directly above the surgical field at a distance of 3050 cm.

Once the patient was ready and draped, the videoendoscope was positioned in the center of the patient’s mouth, while the exoscope was clothed with a sterile cover and positioned directly above the surgical field in a distance of 30–50 cm in order to have enough space for instrument handling (Figure 2). The main 3D monitor (55″) was placed beside the operating table toward its end and directly in front of the first surgeon, while a secondary 3D monitor was set in front of the second surgeon. The first surgeon stood at the patient’s head, facing the monitor. The second surgeon sat behind, using the controller (joystick) and maintained the focus of the camera on the surgical field, adjusting the optical magnification. All surgeons and nurses wore 3D passive polarized glasses, so that the entire surgical team could benefit from the presence of 3D vision during the execution of the procedure.

We used the coblator as an operative instrument; we chose to perform surgery with EVac 70 Xtra HP^®^ as the coblation wand for either ablation or resection, which was used also for tongue base surgery and tontillectomy, which was executed before the lateral pharyngoplasty. It was used at a power of 7 ablation/5 coagulation.

**Figure 3:**
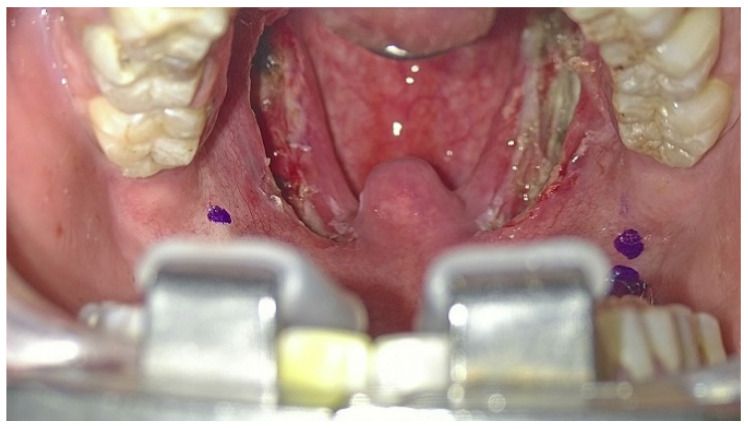
Palatal operative field visualized with the exoscope: the first step of BRP surgery is tonsillectomy, saving as much of the muscular component of the lateral walls of the pharynx as possible.

**Figure 4:**
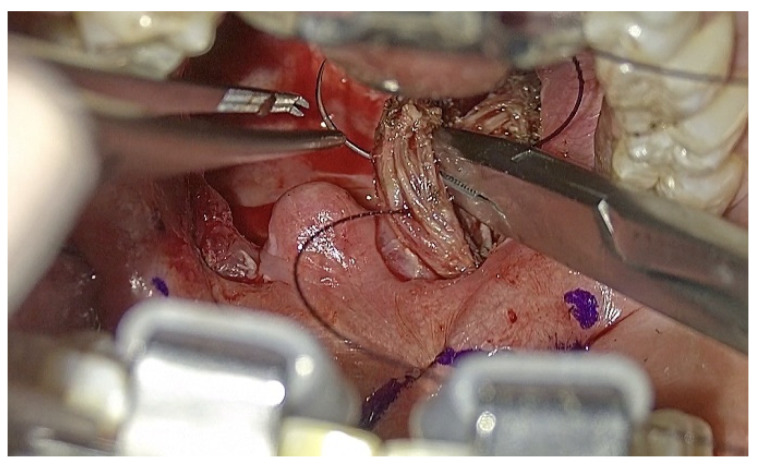
One of the most important step of BRP surgery: the needle must be introduced, from the lateral to the medial regions, posterior to the palato-pharyngeal muscle bundle, which is most commonly at the junction between the superior third and the inferior two thirds of it. The technique requires a second passage at the back, lateral to the raphe and the application of proper tension to the suture in order to reposition the palatopharyngeal muscle more laterally and more anteriorly.

Patients underwent the ablation of 1 cm of tongue base lymphatic tissue on each side of the midline split (2 cm width and 1 cm depth of tissue ablation). The margins of resection include the anterosuperior sulcus terminalis, lateral amygdalo-glossus sulcus and posteroinferior glosso-epiglottic sulcus. Then, the ablation of each palatine tonsil was meticulously realized (Figure 3), sparing the palatopharyngeus muscles and the utmost mucosa covering both pillars in order to perform Barbed Reposition Pharingoplasty. The whole operating room team could see the surgical steps on 3D monitors (Figure 4).

## 3. Results

Currently, seven patients affected by OSA underwent 3D surgery with BRP associated with transoral coblation tongue base reduction and nose surgery. The median age was 53 years (range 40–66), and the median preoperative apnoea–hypopnea index (AHI) and body mass index (BMI) were 30.7 (2536) and 28.9 (23.7–31.5), respectively. The median preoperative Epworth Sleepiness Scale (ESS) score was 10 (6–14). The median 3D system setting time was 12.5 ± 2.3 min. The overall procedure time was 59.3 ± 26 min.

No patients experienced intra-operative, post-operative or delayed complications.

Only one patient experienced a transient dysphagia that spontaneously resolved within one month.

## 4. Discussion

Preliminary evaluations allow us to make some important considerations.

No significant differences were found in the setup times and preparation of the room, both in case of the use of the robot and the coblator (despite the initial difficulties related to the use of a new surgical instrument compared to those of the robot, which has been used for years). The robotic operating room setup times briefly include: docking (the patient side cart was moved to the edge of the patient bed and aligned at a 30° angle from the long axis of the patient surgical bed); exposure of the operative field with Crowe–Davis retractor in order to place the robotic arms in the patient’s mouth: the camera’s endoscope arm was positioned in the center of the patient’s mouth, while the right and left instrument arms were the operative tools used for tissue dissection.

Even the exposure qualities in order to optimally operate are not significantly different despite the two different methods. The 3D visualization of the robot makes it possible to acknowledge small details, such as vascular and nerve structures, which would be difficult to see with the naked eye, allowing a precise and sometimes almost completely bloodless resection to be performed. The quality of visualization using the 4K-3D endoscope, on the other hand, allows the optimal assessment of the depth of field, enabling the surgeon to be attentive and accurate in the resection, despite the initial discomfort/difficulty encountered in coordinating the visualization of the monitor in front of them and gestures linked to the use of the coblator via the transoral route. The similar quality of exposure and visualization means that the resection times are comparable between the two sides, averaging around 10 min for the removal of a lingual tonsil.

The differences between the two approaches are mainly related to the introduction of a new surgical instrument, such as the coblator and the 3D 4K endoscope/exoscope, in the operating field. Therefore, it was harder to prepare the operating field and performing the resection of the baselingual lymphatic tissue, especially for the first patients. It should also be considered, as already mentioned, the initial difficulties related to the innovative use of the coblator on the base tongue and the correct coordination between visual feedback, while the operator’s head is extended in order to correctly view the surgical field on the monitor in front of them with 3D polarized glasses, as well as tactile feedback, which is given via the direct contact between the surgeon and the oropharyngeal district. With the robot, as we know, there is no direct contact between the first operator and the patient.

Other major differences were found considering the extent of resection, which according to a first subjective judgement of the surgeon, was greater for the robot. As a matter of fact, robotic baselingual resection has become by now a standard procedure: the identification of the midline of the superior (terminal sulcus), lateral (amygdala-glossal sulcus) and inferior margins (glossoepiglottic sulcus). With both procedures, a similar number of small intraoperative bleeding events occurred, which were slightly more common when they were performed using the coblator (about 3 on average versus 12 bleedings, or sometimes, complete exsanguination), and which could be easily managed by the second operator handling bipolar forceps, while using the robot, or letting the bleedings clot, while using the coblator.

This allows us to underline further differences between the two systems, as well as the need in robotic surgery for two operators to work together during the surgical procedure. As mentioned above, the second operator has a very important role in order to eliminate any issues that may arise by using robotic arms and to aspirate fumes that may obstruct the view of the first surgeon or to control small intraoperative bleedings with bipolar forceps.

The last difference is the possibility with the robot to obtain samples that can be histologically evaluated, contrary to the coblator, whose main task is linked to the production of bioproducts that determine cellular destruction and allows the tissue resection to be macroscopically performed. This leads us to understand how much more useful the coblator can be in functional surgery compared to neoplastic pathology in which the production of an operative piece is fundamental for the performance of a histological examination.

A very important aspect that should not be overlooked is the difference in the costs between the two technologies. The price of the Da Vinci Robot is around EUR 2,000,000, with annual maintenance costs of around EUR 200,000; each intervention, depending on the type, has a cost that varies from EUR 4500 to 6500. The endoscope + exoscope 4K-3D system has a much lower price, around EUR 200,000, with an additional EUR 20,000 for the purchase of the coblator and an intervention’s cost of less than EUR 1000. Furthermore, the possibility of using 4K-3D technology not only for rhonchi surgery, but also for salivary gland [5], thyroid and ear surgeries in ENT pathology [6,7], as well as laparoscopic approaches in general surgery and gynecology [8], makes it possible to quickly bring down the initial purchase costs.

Finally, the possibility of using Vitom in palatal surgery, although it may seem to be “excessive” and expensive, fulfils an important didactic function: it allows the whole surgical team to visualize a very deep and dark anatomical region, such as the soft palate. Everyone can, therefore, follow the fundamental steps of isolating the palate-pharyngeal muscle and anchoring it to the pterygomandibular raphe on a 3D monitor using polarized glasses in order to stabilize the lateral walls of the pharynx.

## 5. Conclusions

In conclusion, the use of the 4K-3D endoscope and coblator for tongue base resectioning and of the 3D exoscope for lateral pharyngoplasty represents an excellent alternative system in multi-level OSA-related surgery that could reduce the costs of robotic surgery. Furthermore, it also can be used to teach and involve everyone in the surgical team and let them become more aware of the various steps of the surgical act.

## Data Availability

Not applicable.
